# Role of the Gastrointestinal Tract Microbiome in the Pathophysiology of Diabetes Mellitus

**DOI:** 10.1155/2017/9631435

**Published:** 2017-09-26

**Authors:** Muhammad U. Sohail, Asmaa Althani, Haseeb Anwar, Roberto Rizzi, Hany E. Marei

**Affiliations:** ^1^Biomedical Research Center, Qatar University, P.O. Box 2713, Doha, Qatar; ^2^Government College University, Faisalabad 38000, Pakistan; ^3^College of Health Sciences, Qatar University, P.O. Box 2713, Doha, Qatar; ^4^Institute of Cell Biology and Neurobiology (IBCN), Italian National Council of Research (CNR), Rome, Italy

## Abstract

The incidence of diabetes mellitus is rapidly increasing throughout the world. Although the exact cause of the disease is not fully clear, perhaps, genetics, ethnic origin, obesity, age, and lifestyle are considered as few of many contributory factors for the disease pathogenesis. In recent years, the disease progression is particularly linked with functional and taxonomic alterations in the gastrointestinal tract microbiome. A change in microbial diversity, referred as microbial dysbiosis, alters the gut fermentation profile and intestinal wall integrity and causes metabolic endotoxemia, low-grade inflammation, autoimmunity, and other affiliated metabolic disorders. This article aims to summarize the role of the gut microbiome in the pathogenesis of diabetes. Additionally, we summarize gut microbial dysbiosis in preclinical and clinical diabetes cases reported in literature in the recent years.

## 1. Introduction

The gastrointestinal tract (GIT) harbors a dense and diverse microbial community, which includes archaea, bacteria, protozoans, and viruses, and is commonly referred to as microbiome. There are approximately 100 trillion bacteria that occupy the GIT mucosal surface, constantly interacting with metabolically and immunologically active cells. These microbes not only act as the first line of defense against foreign particles but also initiate a vast array of immunological activities that augment mucosal and systemic immunity [1]. The GIT microbiome displays very diverse physiological features: digestion of complex carbohydrates, vitamins synthesis, immune and inflammatory response modulation, and hormones and neurotransmitter production [2]. The much-emphasized gut-brain axis and gut-hypothalamus axis are influenced by microbes through unknown methods/factors to regulate food intake, metabolism, and energy homeostasis [3]. Through these neuronal and endocrine axes, microbes can sense host metabolic status and alter nutrient availability to meet the body needs. Brown and Hazen [4] described the GIT microbiome as an endocrine organ that translates nutritional cues into hormone-like signals to impact host physiology and diseases. Recently, there have been several scientific reports that link the GIT microbiome with systemic diseases including obesity, diabetes, hepatopathy, rheumatoid arthritis, cancer, and cardiovascular diseases [5–8].

Diabetes mellitus is an important metabolic disorder of public health significance that results from a myriad of factors. A recent survey suggests that approximately 422 million individuals around the globe suffer from diabetes and, by 2030, diabetes will be the 7th leading cause of human death [9]. The current global prevalence rate of diabetes is 8.5%, and the estimated death toll for direct diabetes-caused morbidity for 2014 was 1.6 million [10]. Perhaps, the figure may get much higher if we also include deaths caused by high blood glucose associated with other diseases. Over time, diabetes can lead to secondary complications, such as cardiovascular disease, cerebrovascular disease, neuropathy, retinopathy, nephropathy, and limb amputation [10, 11]. Broadly, diabetes is categorized among two common types, insulin-dependent type 1 diabetes mellitus (T1DM) and insulin-independent type 2 diabetes mellitus (T2DM). The less common types of diabetes include gestational diabetes, monogenic diabetes (inherited form), and cystic fibrosis-related diabetes. Among all of these types of diabetes, the former two are the most discussed conditions and have slightly different etiologies and pathogenesis, but mainly common outcomes.

Genetics, nutrition, autoimmunity, and the environment may be few of the many etiological factors that partially or collectively contribute to the diabetes disease pathology. Among the environmental factors, the GIT microbiome has gained much interest, based in part on experimentation in human diabetic subjects and nonobese diabetic (NOD) mice or biobreed diabetes rodent models [12]. Studies with the germ-free model of NOD mice reported enhanced susceptibility to autoimmune and allergic diseases and spontaneous development of diabetes [13]. In brief, accumulated evidences suggest a prominent role of microbiome in diabetes, autoimmunity, and other metabolic diseases. The present article, therefore, is designed to delineate the significance of GIT microbiome in the pathophysiology of diabetes and how management of microbiome can relieve the patient.

## 2. Role of GIT Microbiome in Host Metabolism and Energy Homeostasis

**Figure 1:**
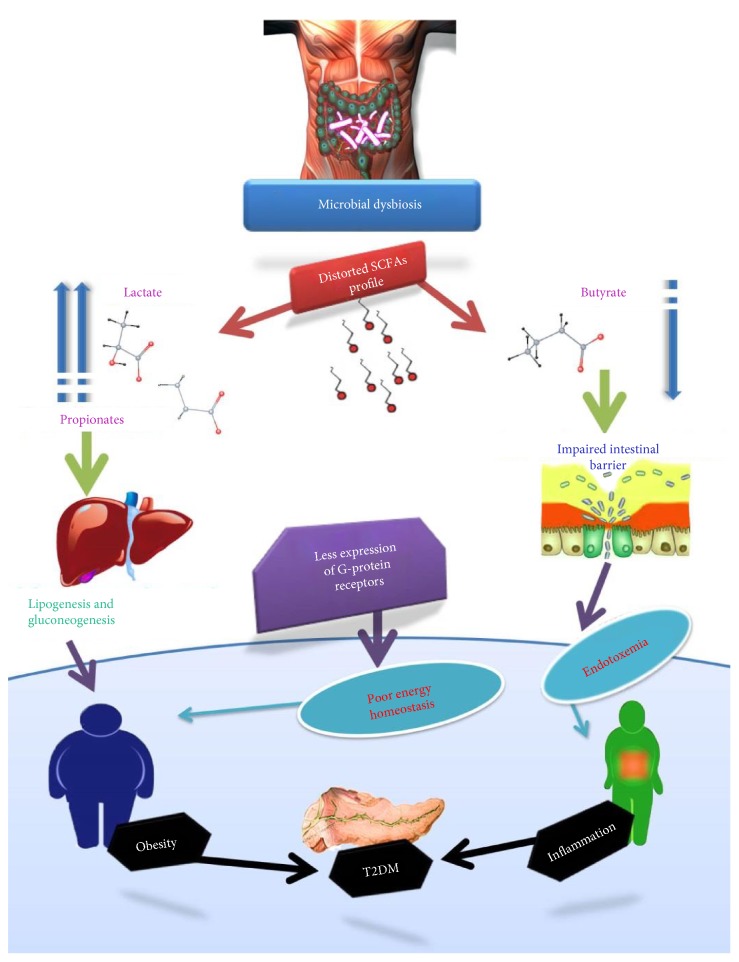
Change in the microbiome fermentation profile changes gut permeability and energy homeostasis which causes endotoxemia, low-grade inflammation, and obesity. Poor energy homeostasis leads to hyperglycemia and hyperlipidemia which may lead to obesity and ultimately insulin resistance.

Hosts and their microbiomes develop symbiotic relationships through interactive evolutionary processes that mutually benefit both. In a broader sense, the resident symbionts regulate host metabolism in multiple ways, integrating physiological homeostasis, immune-inflammatory signaling, and energy compliance. Multiple mechanisms are thought to link microbial activity in the GIT and the systemic metabolism. Carbohydrates are the primary sources of energy for both the human host and their microbes. Conventionally reared rodents have higher carbohydrate metabolites from glycolysis and tricarboxylic acid cycle compared to germ-free rodent models demonstrating that conventionally reared rodents have a higher energy-harvesting capability [14]. Humans lack enzymes for digestion of complex carbohydrates, including cellulose, resistant starch, xylans, and inulin. In contrast, the microbiome encodes enzymes required for indigested carbohydrate fermentation. Microbial fermentation harvests energy for microbial growth and generates monosaccharides and short-chain fatty acids (SCFAs). The SCFAs act as ligands for the G protein-coupled receptors GPR41 and GPR43, expressed by enteroendocrine cells in the GIT mucosa (Figure 1) [15–17]. Hooper et al. [18] observed that colonization of germ-free rodents with GIT microbes obtained from conventional rodents induced sodium/glucose cotransporter-1 expression in epithelial enterocytes. Specifically, inoculation of *Bacteroides thetaiotaomicron* in humans and mice promoted expression of genes involved in nutrient absorption, mucosal barrier integrity, angiogenesis, and xenobiotic metabolism [18, 19].

Short-chain fatty acids have significant effects on the GIT wall health as, for example, a source of energy, anti-inflammation agents, angiogenics and vasodilators, promotility agents, and wound healing agents [20]. Microbial fermentation products also affect the muscles, liver, brain, and adipose tissue metabolism. The liver metabolic profile of gnotobiotic mice is different from that of conventionally raised mice, probably because of an over influx of SCFAs into the liver. Both hepatocytes and enterocytes are reported as energy deprived and have an overexpression of AMP-activated protein kinase (AMPK), which determines cellular energy status in gnotobiotic germ-free mice [21, 22]. Butyrate is principally used as an energy source for enterocytes, whereas acetate and propionate are flushed to the liver for lipogenesis and gluconeogenesis. Butyrate supplementation to obese, prediabetic mice significantly improved the intestinal epithelial barrier and insulin secretion from beta cells and decreased body adiposity as well as weight gain, insulin resistance, hyperinsulinemia, and hyperglycemia [23]. Everard et al. [24] reported that prebiotic, functional foods that alter the microbiome fermentation profile improve energy balance and leptin sensitivity by modulating enteroendocrine cell secretions in obesity and diabetic mice models. Moreover, prebiotic supplementation reduces hunger, increases satiety, and decreases total energy intake by about 10% [25]. In the last few decades, *Lactobacillus* has gained much importance as probiotic, live microbes that augment the microbial profile. Li et al. [26] found that *Lactobacillus* supplementation improved insulin homeostasis accompanied by glucose tolerance and protection of beta cell islets in diabetic mice.

Notably, certain bacterial clades, for example, Bacteroidetes and Firmicutes, enhance ATP-binding cassette transporter expression in enterocytes and glucagon-like peptide 1 and 2 secretion. Everard et al. [24] observed a positive correlation between genus *Anaerotruncus* abundance in the GIT and gut permeability, glucose intolerance, blood triglyceride content, and muscle lipid concentrations. Similarly, family *Desulfovibrionaceae* is associated with dyslipidemia and obesity [27]. In a broader sense, a microbiome shift, delineated by a rise in Firmicutes and a decline in Bacteroidetes populations, is implicated in obesity. The underlying mechanisms for these interactions are not yet fully understood. Although the aforementioned literature enhances our understanding of the role of the microbiome in host metabolism and energy homeostasis, the identification of better molecular markers of metabolism regulation is of greater significance.

## 3. GIT Microbiome and Metabolic Disorders as Precursors to Diabetes

**Figure 2:**
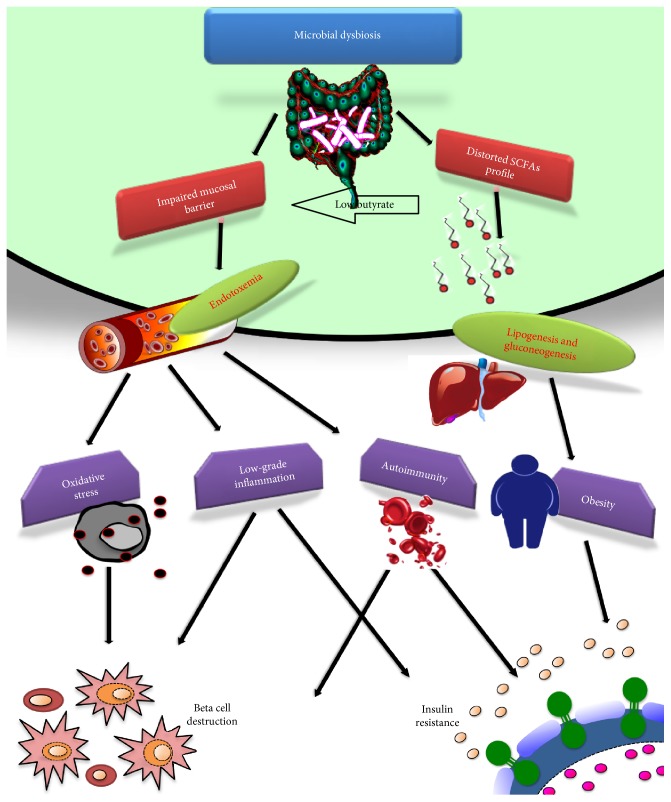
A schematic diagram describing the role of the gastrointestinal tract microbiome in the development of the metabolic syndrome that leads to diabetes mellitus pathogenesis. Microbial dysbiosis impairs intestinal wall integrity and allows translocation of toxins from the gut lumen to the systemic circulation. This endotoxemia leads to low-grade inflammation, autoimmunity, and oxidative stress that may lead to beta cell destruction or insulin resistance.

The gastrointestinal tract microbiome interacts with host nutrition, the environment, and host genetics for the development of obesity-related metabolic disorders. Various studies have reported that GIT microbial dysbiosis enhances energy harvest and expression of obese phenotype. High throughput amplicon sequencing of the 16S rRNA gene reveals that a change in the Bacteroidetes/Firmicutes ratio is associated with higher expression of microbial genes that encode enzymes related to carbohydrate metabolism. The microbiomes of obese persons differ from those of lean individuals and, generally, are characterized by a lower prevalence of phylum Bacteroidetes and a higher prevalence of phylum Firmicutes [28]. Therefore, changes in the Bacteroidetes/Firmicutes ratio portray an environmental factor that provides genetic material for increased capacity to harvest energy from the diet [29]. The higher energy harvest promotes lipogenesis and increases the number and size of lipid droplets in the extraintestinal tissues (Figure 2). Most patients suffering from this metabolic syndrome have excessive fat accumulation which suggests that the dyslipidemia is an important etiological factor of the syndrome [30].

Autoimmunity, insulin resistance, and hypertension are a few potentially lethal consequences of obesity. Transient changes in the microbiome can disrupt the microbiome and host-immune axis. There is an increasing amount of evidence that suggests that intestinal commensals directly influence the development of autoimmunity and low-grade inflammation [31]. In general, the inadequately functional immune system of gnotobiotic mice or neonates suggests that its maturation is compelled by the resident microbiome. However, cellular and molecular processes by which GIT microbes promote autoimmune responses are poorly understood. Different studies propose more than one method of immune disruption in systemic and local immune systems in response to changes in microbial ecology. *Candidatus savagella*, a normal commensal bacterium, is associated with the development of autoimmune arthritis and encephalomyelitis. In contrast, the same bacterium is involved in protection against autoimmune T1DM [32]. In the previously mentioned experiments, excessive production of T helper 17 (Th-17) by *Candidatus savagella* has a causal role in autoimmune diseases. In other studies, commensal *Bacteroides fragilis* has been associated with systemic Th1 cells and local interleukin-10- (IL-10-) producing regulatory T cells [33]. The microbe can prevent autoimmune encephalomyelitis in mice via conversion of naïve CD4+ T cells into IL-10-producing regulatory T cells [32]. In addition, SCFAs, through G protein-coupled receptors, have been associated with inflammatory bowel disease, colitis, arthritis, diabetes, and asthma [34].

Microbiome-triggered chronic low-grade inflammation is another important causal factor for obesity and related metabolic syndromes. Cani et al. [35] found that metabolic endotoxemia, caused by extraintestinal lipopolysaccharides (LPS) infiltration, causes inflammation, oxidative stress, obesity, and diabetes. Resident Gram-negative bacteria secrete LPS and other endotoxins in the GIT. These endotoxins can cross the GIT mucosal barrier through mucosal tight junctions or by infiltrating chylomicrons [36]. Once in the extraintestinal tissue, endotoxins trigger innate immune responses by activating CD14, nucleotide oligomerization domain (NOD), and toll-like receptor 4 (TLR4) at the surface of dendritic cells and macrophages. Furthermore, recent studies suggest activation of innate immune system by recruitment of effector molecules (inflammasomes, peptidoglycans, TNF-*α*, IL-1*β*, and flagellin) in response to LPS infiltration [37]. In brief, microbial dysbiosis, caused by high-fat diet supplementation, can increase intestinal permeability, LPS infiltration, oxidative stress, macrophage infiltration, and adipose tissue inflammation [35].

## 4. Type 1 Diabetes Mellitus

Type 1 diabetes is characterized by a lack of sufficient insulin production and elevated blood glucose levels. The disease mostly occurs in children and young adults and, therefore, is also called juvenile diabetes. The disease is usually caused by oxidative and/or T cell-mediated autoimmune destruction of pancreatic beta cells, leading to partial or complete loss of insulin production [38, 39]. The specific environmental constituents eliciting beta cell autoimmune destruction are not fully known. It is thought that in hereditarily prone persons, a chronic inflammatory disease of the GIT elicits the primary offense, leading to autoimmune destruction of *β*-islet cells [40]. Recent research has highlighted the role of the GIT microbiome in the development of T1DM and that the disease is associated with unidentified GIT microbiome dysbiosis [41]. Brown et al. [42] found a higher proportion of Actinobacteria, Bacteroidetes, and Proteobacteria phyla in TIDM subjects, whereas, the control group had higher abundance of Firmicutes, Fusobacteria, Tenericutes, and Verrucomicrobia. A longitudinal study on young children suggested that bacterial diversity diminishes over time in genetically prone autoimmune children when compared with healthy control age-mates. Particularly, it was observed that *Bacteroides ovatus* contributed almost 24% of constituents in the phylum Bacteroidetes in the T1DM subjects [43]. Results from other studies [44–46] have also shown changes in the microbial ecology and a decline in bacterial diversity in T1DM cases. However, there is still a scarcity of scientific literature that connects such alterations in the microbiome as a predictor of its functional role in the disease pathology (Table 1).

The available literature suggests that there might be more than one coincident or independent pathogenesis route through which the GIT microbiome can lead to beta cell destruction and the onset of T1DM. In the first concept, as mentioned above, microbiome dysbiosis-associated immune regulation may lead to T cell-mediated destruction of beta cells in genetically susceptible individuals [35, 47]. The second proposed pathogenesis correlates T1DM with gut leakiness, endotoxemia, and immune-deregulation-associated chronic low-grade inflammation [1, 36]. Some bacterial species, such as *Akkermansia muciniphila*, *Bacteroidetes thetaiotaomicron*, and *E. coli*, enhance gut permeability and endotoxemia which may lead to metabolic syndrome [48].

Through earlier experiments, it is widely recognized that oxidative stress plays a major role in the development of diabetes. A comparatively less-discussed notion is the metabolic-related oxidative stress under microbial dysbiosis. Morgan et al. [49] observed microbial dysbiosis and disrupted nutrient availability during metabolic syndromes associated with major changes in oxidative stress pathways. Furthermore, supplementation of *Lactobacillus-*based probiotic yogurt has been shown to suppress streptozotocin-induced diabetogenic state and to improve antioxidant status [50].

It is still uncertain if these microbiome-triggered oxidative stress, autoimmunity, or inflammatory abuses of beta cells act independently or work together. Perhaps, underlining mechanisms have interrelated pathways and common output.

## 5. Type 2 Diabetes Mellitus

Hyperglycemia associated with progressive resistance to insulin action and inappropriate insulin secretion is characterized as T2DM. The disease usually develops in genetically susceptible adults due to a sedentary lifestyle and represents as much as 90% of all the cases of diabetes. Over the past few decades, the disease incidence has increased tremendously along with an early age onset. The etiology and pathogenesis of the T2DM are a very complex assembly of genetic and epigenetic elements influenced by a complex societal framework and environmental factors. Among the environmental factors of T2DM, GIT microbiome assessment and characterization have attracted major research interest in the recent years. Metformin is the most commonly used medication for controlling hyperglycemia in T2DM patients. The exact mode of action of this drug is not well defined, though it has been shown that the drug improves gut microbial diversity, metalloproteins-encoding gene expression in gut bacterial species, and glycemic index [51].

The gut microbiome interacts with host genetics and encodes several essential proteins required for human health and disease [52]. Xu and Gordon [53] suggested that microbes can modify gene expression in the host to create physiological homeostasis or to elicit certain disease pathogenesis. Particularly, microbiome-triggered changes in intestine tight-junction proteins and alkaline phosphatase activity in the gut milieus may increase gut permeability and lead to the pathogenesis of insulin resistance [54]. In obese individuals, T2DM pathogenesis is linked to activation of inflammatory pathways that cause insulin resistance through activation of the I*κ*B kinase complex, protein kinases 1 and 2, and c-Jun N-terminal kinases (JNKs) in the tissues [55]. In brief, the predominant notion of T2DM pathogenesis incorporates a nexus of microbial dysbiosis, gut leakiness, autoimmunity, chronic inflammation of adipose tissue, obesity, and insulin resistance [56].

Microbial dysbiosis associated with T2DM is characterized by poor species richness and diversity (Table 1). Quite a few studies reported changes in the abundance of certain bacterial clades in diabetic conditions, such as a change in the Firmicutes/Bacteroidetes ratio [44]. Correction of microbial dysbiosis by supplementation with prebiotics, as reported by Cani et al. [36], improved *Bifidobacterium* abundance which is significantly and positively correlated with improved glucose tolerance and inflammation in prebiotic-treated mice. Similarly, Wu et al. [57] also reported a higher abundance of *Bifidobacterium* in healthy subjects when compared with T2DM patients. Furthermore, a T2DM metagenome study revealed an enrichment of sulphate reduction and oxidative stress resistance functions and a decline in some butyrate-producing bacteria in T2DM patients [46]. Short-chain fatty acids produced during microbial fermentation, particularly butyrates, enhance gut wall integrity and prevent metabolic endotoxemia, inflammation, and associated disorders.

Currently, there is very little published work that characterizes the microbiome and metagenomics of diabetes. Additionally, the published literature shows contradictions in taxonomic affiliations of the microbiome with diabetes. Furthermore, most of the published work explored only phylogenetic characteristics of the diabetes microbiome and pays far less attention to the functional contents of the microbiome. Perhaps, based on the available literature, it can be concluded that changes in the microbiome may have substantial effects on host metabolism [29]. Any significant change in bacterial diversity may affect the gut fermentation profile, gut wall permeability, and mucosal and systemic immunity and, therefore, may trigger mechanisms responsible for oxidative stress, endotoxemia, obesity, and hyperglycemia [5]. While many reports have shown changes in the ratio of the predominant phyla in the gut, the question remains as to the functional effects of such constituents and if this are clinically significant.

## Figures and Tables

**Table 1:** Summary of the gut microbiome and metagenomic changes observed in different preclinical and clinical diabetes studies.

Study format	Clinical features/major findings	Microbiome changes	Metagenome/metabolome changes	Reference
Longitudinal infant T1DM	Out of 33 genetically predisposed T1DM infants, 12% developed T1DM, whereas 21% developed T1DM autoantibodies during the first 4 years of life	A decrease in alpha diversity and an overabundance of Blautia and Rikenellaceae	Modulation of sphingomyelin, lithocholic acid, lipids, branch-chained amino acid concentrations, and sugar transport pathways	[55]

Longitudinal infant T1DM	All four enrolled infants developed autoimmunity and T1DM within the first 3 years	Drop in alpha diversity and increase in Bacteroidetes (*Bacteroides* spp.) and decrease in Firmicutes	Not applicable	[43]

Metagenomics of the microbiome in T1DM patients	Microbial fermentation and functional components promoted autoimmune destruction of beta cells	Higher Bacteroides and lower *Prevotella* abundance	T1DM patients had higher carbohydrate metabolism, adhesions, motility, phages, prophages, sulfur metabolism, and stress responses	[42]

Metagenomics of the microbiome in T2DM patients microbiome	Not applicable	Microbial dysbiosis characterized by a decrease in butyrate-producing bacteria and an increase in the populations of various opportunistic pathogens	Higher gut oxidative stress and membrane transport of sugars	[46]

Metagenome in T2DM women	Elevated glucose, C peptide, leptin, triglycerides, and oxidative stress	Enriched with *Lactobacillus* sp. and depletion of *Clostridium* sp.	Higher sugar metabolism and transport, fatty acid synthesis, and oxidative stress pathways	[58]

Adult T2DM	Ratio of Bacteroidetes to Firmicutes correlated positively and significantly with plasma glucose concentrations	Higher alpha diversity. Changes in beta diversity were characterized by higher Bacteroidetes in T2DM cases and Firmicutes belonging to class Clostridia in controls subjects	Not applicable	[44]

Metagenomics of T2DM patients before and after bariatric surgery	Surgery improved BMI, hypertension, lipid profile, and glycemic index	Bacteroidetes/Firmicutes ratio increased. Several changes in taxonomy composition	Changes in carbohydrate metabolism and the phosphotransferase system	[59]

Antibiotic treatment in high-fat diet-induceddiabetic mice	Antibiotic treatment reduced endotoxemia, glucose intolerance, body weight gain, inflammation, and oxidative stress	Antibiotic treatment changes microbiome architecture of high-fat diet-induced diabetic mice	Drop in endotoxemia, tissue inflammation, and oxidative stress markers	[35]

Fecal transplant from healthy mice to T1DM genetically susceptible mice	Prevents autoimmunity, and insulitis and delays T1DM development	Increase in Bacteroidetes and decrease in Firmicutes and *Clostridiaceae* and *Lactobacillaceae* abundance	Increase in IgA, TGF*β* concentrations, and CD8+, CD103+, and CD8*αβ* T cells	[60]

Antibiotic treatment of biobred diabetes-prone rat	Antibiotic treatment delayed/protected against TIDM	Antibiotic treatment lowered *Bacteroides* spp.	Antibiotic treatment lowered insulitis	[12]
